# Exploring the Role of ACE2 as a Connecting Link between COVID-19 and Parkinson’s Disease

**DOI:** 10.3390/life13020536

**Published:** 2023-02-15

**Authors:** Efthalia Angelopoulou, Eleni Karlafti, Vasiliki E. Georgakopoulou, Petros Papalexis, Sokratis G. Papageorgiou, Thomas Tegos, Christos Savopoulos

**Affiliations:** 1 Department of Neurology, Aiginition University Hospital, National and Kapodistrian University of Athens, 11527 Athens, Greece; 2 1st Propaedeutic Department of Internal Medicin, AHEPA University General Hospital, Aristotle University of Thessaloniki, 54124 Thessaloniki, Greece; 3 Emergency Department, AHEPA University General Hospital, Aristotle University of Thessaloniki, 54124 Thessaloniki, Greece; 4 Department of Infectious Diseases-COVID-19 Unit, Laiko General Hospital, 11527 Athens, Greece; 5 Unit of Endocrinology, First Department of Internal Medicine, Laiko General Hospital, Medical School, National and Kapodistrian University of Athens, 11527 Athens, Greece; 6 Department of Biomedical Sciences, University of West Attica, 12244 Athens, Greece; 7 First Neurology Department Medical School, AHEPA University Hospital, Aristotle University of Thessaloniki, 54124 Thessaloniki, Greece

**Keywords:** neurodegeneration, dopaminergic degeneration, neuroinflammation, COVID-19 pandemic

## Abstract

Coronavirus disease 2019 (COVID-19) is frequently accompanied by neurological manifestations such as headache, delirium, and epileptic seizures, whereas ageusia and anosmia may appear before respiratory symptoms. Among the various neurological COVID-19-related comorbidities, Parkinson’s disease (PD) has gained increasing attention. Some cases of PD disease have been linked to COVID-19, and both motor and non-motor symptoms in Parkinson’s disease patients frequently worsen following SARS-CoV-2 infection. Although it is still unclear whether PD increases the susceptibility to SARS-CoV-2 infection or whether COVID-19 increases the risk of or unmasks future cases of PD, emerging evidence sheds more light on the molecular mechanisms underlying the relationship between these two diseases. Among them, angiotensin-converting enzyme 2 (ACE2), a significant component of the renin-angiotensin system (RAS), seems to play a pivotal role. ACE2 is required for the entry of SARS-CoV-2 to the human host cells, and ACE2 dysregulation is implicated in the severity of COVID-19-related acute respiratory distress syndrome (ARDS). ACE2 imbalance is implicated in core shared pathophysiological mechanisms between PD and COVID-19, including aberrant inflammatory responses, oxidative stress, mitochondrial dysfunction, and immune dysregulation. ACE2 may also be implicated in alpha-synuclein-induced dopaminergic degeneration, gut–brain axis dysregulation, blood–brain axis disruption, autonomic dysfunction, depression, anxiety, and hyposmia, which are key features of PD.

## 1. Introduction

Coronavirus disease 2019 (COVID-19) emerged in December 2019 as a global pandemic, causing a significant health threat worldwide [1]. COVID-19 is caused by the severe acute respiratory syndrome coronavirus 2 (SARS-CoV-2). It is well documented that SARS-CoV-2 uses angiotensin-converting enzyme 2 (ACE2) in order to enter host cells [1]. ACE2 is a significant component of the renin-angiotensin system (RAS), which is critically involved in the cardiovascular system, but also plays a major role in the regulation of inflammatory responses in multiple tissues, including the brain [2].

More specifically, reduced ACE2 expression and activity have been shown in hypertension, heart failure, atherosclerosis, diabetic nephropathy, and other disease models. Local inhibition or total ablation of ACE2 in the brain reduces baroreflex sensitivity [3]. Furthermore, ACE2-null animals have been demonstrated to suffer either high blood pressure or cardiac dysfunction [3]. ACE2 overexpression, on the other hand, has protective effects on local tissues, including the brain. ACE2 is found throughout the brain, including nuclei involved in the central control of cardiovascular function, such as the brainstem’s cardio-respiratory neurons, as well as non-cardiovascular regions, such as the motor cortex and raphe. The existence of ACE2 mRNA and protein in the mouse brainstem has also been verified. While these data indicate that ACE2 is a novel component of the brain RAS, they also reveal that ACE2’s participation in the central nervous system extends beyond the control of cardiovascular function [3].

COVID-19 is frequently accompanied by neurological manifestations such as headache, delirium, and epileptic seizures, whereas ageusia and anosmia may appear before respiratory symptoms [4]. In COVID-19 patients, SARS-CoV-2 has been detected in the cerebrospinal fluid and cortical neurons in post-mortem brain biopsies, suggesting that the virus might directly affect neuronal tissue [5]. A study in human brain organoids demonstrated that SARS-CoV-2 could infect neurons, and ACE2 inhibition prevented this process [1]. COVID-19 has been associated with increased microglial activation in humans and animal models. Nevertheless, there is still a debate over whether the neurological symptoms of COVID-19 are caused by the direct invasion of the virus in the brain or the inflammatory consequences of the infection [6].

The association between COVID-19 and hypertension, cardiovascular disorders, cerebrovascular disease, and chronic kidney disease has been extensively investigated and reviewed [7]. Among the various neurological COVID-19-related comorbidities, Parkinson’s disease (PD) has gained increasing attention.

PD is the second most common neurodegenerative disorder, affecting approximately 2% of the population over the age of 60 [8]. It is characterized by motor symptoms, such as resting tremor, rigidity, and bradykinesia, as well as non-motor symptoms, such as psychosis, depression, hyposmia, autonomic dysfunction, and cognitive impairment [8,9]. PD is characterized as a multifactorial entity, since both genetic factors and environmental components contribute to its pathogenesis. Emerging evidence highlights the key roles of neuroinflammation, immune responses, mitochondrial impairment, dysregulation of apoptosis, impaired gut microbiome homeostasis, and pathogens in its pathophysiology. However, the etiology of PD remains elusive, and there is still no available treatment that could halt disease progression [10].

COVID-19 has been associated with significant deterioration of motor and non-motor manifestations in PD patients [10]. Older age and a longer duration of the disease may increase susceptibility to SARS-CoV-2 infection in PD patients [11]. A recent systematic review demonstrated no significant difference in COVID-19-related mortality and hospitalization in PD patients compared to non-PD individuals [12].

It is still unclear whether PD increases the susceptibility to SARS-CoV-2 infection or whether COVID-19 increases the risk or unmasks future cases of PD. The hypothesis that COVID-19 may increase PD risk is also based on historical data reporting parkinsonian symptoms caused by encephalitis lethargica, a disease that affected over one million individuals between 1916 and 1930 and is characterized by high fever, sore throat, headache, lethargy, double vision, delayed physical and mental response, sleep inversion, and catatonia. The causes of encephalitis lethargica remain unknown. Some research has investigated its origin in an autoimmune response as well as connections to the pathologies of infectious disease—viral and bacterial [13]. There are some cases of parkinsonism related to COVID-19 that occurred shortly after or concurrently with the infectious disease, representing potential post-viral parkinsonian syndromes. Some of these cases also showed evidence of nigrostriatal pathway deficits in the functional imaging, suggesting potential dopaminergic degeneration [14].

Excessive inflammatory responses, immune dysregulation, oxidative stress, mitochondrial dysfunction, and aging are some strong links connecting COVID-19 and neurodegenerative diseases, including PD [15,16]. However, the molecular mechanisms underlying the relationship between PD and COVID-19 still remain elusive.

A growing body of evidence suggests that ACE2 plays a key role in the pathogenesis of both COVID-19 and PD, via its implication in the shared underlying mechanisms of these diseases: inflammation, immune responses, oxidative stress, cell proliferation and survival, and mitochondrial function. Although the role of ACE2 in neurodegenerative diseases and COVID-19 has been previously discussed [17,18,19], there has been no recent extensive review focusing on the latest evidence, particularly for PD. Given the rapidly growing body of research in this field, we intend to provide an updated critical review on the relationship between COVID-19 and PD in particular, using ACE2 as a potential connecting link between these two conditions. In addition, based on the shared pathophysiological mechanisms of these two diseases, we provide insights for future research and the development of ACE2-related targeted treatment approaches.

## 2. ACE2: Structure, Activity, and Biological Functions

RAS was first described in 1898, and it has been considered that it contributed to the adaptive process from the aquatic to the terrestrial ecosystems [20]. Renin, a protease, cleaves angiotensinogen to produce angiotensin I [21]. Angiotensin-converting enzyme (ACE), a metalloprotease, cleaves angiotensin I to produce angiotensin II. Angiotensin II exerts several cellular functions via its two G-protein-coupled receptors: angiotensin II type 1 receptor (AT1R) and angiotensin II type 2 receptor (AT2R) [21]. AT2R usually counter-regulates the activity of AT1R [22]. Apart from angiotensin I and II, additional angiotensin peptides have been recognized, including angiotensin (1–7), angiotensin III, angiotensin IV, angiotensin A, and alamandine [23,24,25,26,27].

About twenty years ago, an ACE homologue, ACE2, was discovered [28]. ACE2, also known as angiotensin-converting enzyme homolog (ACAH), metalloprotease 15 (MPROT15), and ACE-related carboxypeptidase, is an 805 amino-acid-long metalloproteinase of the M2 family [21]. While the gene encoding ACE is located on chromosome 17 [29], the gene encoding ACE2 is located on the X chromosome [30]. ACE2 is a type I transmembrane glycoprotein, and it consists of two domains: the carboxy-terminal domain and the amino-terminal domain, which includes the catalytic site. The active site of the catalytic domain of ACE2 is exposed to the extracellular space, enabling its interaction with various circulating peptides. The catalytic activities of ACE2 in its active site are mediated by zinc, and histidines coordinate them [21]. Similar to ACE, the activity of ACE2 is also modulated by chloride ions [21,31]. In the metalloprotease catalytic domains, ACE2 has 42% sequence similarity with ACE; however, unlike ACE, the carboxypeptidase hydrolyzes its substrates by removing a single amino acid from their respective C-terminal. The decapeptide angiotensin I and octapeptide angiotensin II may be cleaved by ACE2 to yield angiotensin (1–9) and angiotensin (1–7), respectively. Because angiotensin I has a low affinity for ACE, the conversion of angiotensin I to angiotensin (1–9) is not physiologically significant, particularly in situations where ACE activity is restricted or angiotensin I levels are elevated. It has been determined that ACE2 has a 400-fold stronger affinity for angiotensin II than angiotensin I. As a result, the primary function of ACE2 in Ang peptide metabolism is the formation of angiotensin (1–7). ACE2 is also involved in the metabolism of non-RAS peptides such as dynorphin, neurotensin, apelin-13, [des-Arg9]-bradykinin, and [Lys-des-Arg9]-bradykinin [3].

ACE2 mainly acts as a carboxypeptidase that degrades peptides, such as angiotensin I and II, on the surface of the cell membrane [29]. Apart from the catalyzing activity of ACE2 via its extracellular domain, its transmembrane domain also exerts cellular functions, including its role in the cell entry of SARS-CoV-2 [32].

ACE2 is primarily expressed in the heart, renal, and testicular tissue, but it is also present at lower levels in several other tissues, especially the gut, liver, lungs, and brain [28,33]. ACE2 functions as a downregulator of RAS, thereby acting in a protective manner in cardiovascular diseases, nephropathies, and acute respiratory distress syndrome (ARDS) in vivo [29,34]. In the renal tissue of patients with diabetic nephropathy, ACE2 and ACE are shown to be downregulated and upregulated, respectively [35].

Regarding the transcriptional regulation of ACE2, physiological and various pathological conditions cause a dynamic change in ACE2 mRNA levels [36]. ACE inhibitors, AT1R blockers, aldosterone inhibitors, the transcription factor nuclear factor kappa B (NF-B), and pro-inflammatory cytokines such as interferon and interleukin-4 (IL-4) have all been shown to increase ACE2 expression [21]. The promoter of ACE2 contains hypoxia-responsive elements, and hypoxic conditions can upregulate ACE2 expression in a HIF1A-independent manner [37]. However, it is still unclear whether hypoxia can induce ACE2 expression in neuronal or glial cells. Epigenetic modifications, such as DNA methylation, post-translational histone modifications, and microRNAs also affect ACE2 expression [38], although their role in PD and COVID-19 remains largely unknown.

## 3. The Role of ACE2 in COVID-19: Mechanistic Insights

SARS-CoV-2 belongs to the betacoronaviruses group [29]. It is an enveloped single-stranded RNA virus, containing approximately 30,000 nucleotides [39]. The genetic content of SARS-CoV-2 encodes four proteins: spike glycoprotein (S), the nucleocapsid protein (N), a small envelope protein (E), and matrix protein (M) [40]. The spike glycoprotein (S) mediates the entry of the virus into the host cells, and it has two subunits, known as S1 and S2 [40].

ACE2 is required for the entry of SARS-CoV-2 into the host cell and, subsequently, the following viral replication [41]. In particular, the RBD of the spike (S) protein of SARS-CoV binds to ACE2, which acts as the functional cellular receptor [41,42]. The serine protease transmembrane protease serine 2 (TMPRSS2) of the host cell is used for S protein priming [43]. Importantly, SARS-CoV could still infect cells expressing mutant forms of ACE2 with no catalytic activity [21]. The spike (S) protein of SARS-CoV could interact with the tip of subdomain I of the catalytic domain of ACE2 without affecting subdomain II or occluding the enzymatic active site [44].

Despite the significant genetic and structural similarities between SARS-CoV and SARS-CoV-2, SARS-CoV-2 displays greater infectivity and transmissibility, which has resulted in the massive and rapid increase in the number of COVID-19 patients [29]. The spike protein of SARS-CoV-2 also exhibits increased binding affinity to ACE2 compared to SARS-CoV [45]. The entry of SARS-CoV2 into the host cells has also been demonstrated to depend on ACE2 and S protein priming by TMPRSS2 [46], suggesting that ACE2 is a main determinant of SARS-CoV-2 entry into the target cells. Importantly, the peptidase activity of ACE2 is separated by the site of ACE2 that binds to the spike protein (S) of SARS-CoV-2, and the binding of S protein to ACE2 seems to not alter the catalytic activity of the virus [46].

In the lung tissue, ACE2 is highly expressed in the alveolar epithelial cell types I and II, where it can facilitate SARS-CoV-2 invasion [47]. However, ACE2 is also expressed in vascular endothelial cells, as well as lung progenitor/epithelial stem cells [48]. This observation might at least partially explain the ability of SARS-CoV to continuously destroy the lung tissue, which is associated with limited repair capacity. The fact that ACE2 is also expressed in the heart, renal, and intestinal tissues [49] may also at least partially explain the multi-organ injury of SARS-CoV2-infected patients [50,51,52,53].

ARDS is a severe complication of SARS coronaviruses infection, characterized by high mortality [54,55,56]. ACE2 has been shown to act in a protective manner against ARDS. ACE2 knockout has been associated with more severe ARDS pathology in mice, accompanied by increased inflammation and lung tissue damage, increased pulmonary edema, and worse respiratory function compared with the wild type [57]. Delivery of recombinant ACE2 protein could also improve the respiratory function in ARDS animal models [58].

The SARS-CoV-2-mediated ACE2 downregulation and subsequent RAS upregulation may also be involved in other cellular pathophysiological mechanisms. Apart from the enhanced inflammatory responses and tissue injury, overactivation of RAS may also result in oxidative stress, mitochondrial impairment, and autophagy dysregulation. In this regard, SARS-CoV-2 infection of the renal tubular epithelium is associated with mitochondrial dysfunction in an ACE2-dependent manner [59]. Inhibition of ACE2 SUMOylation has also been shown to protect against SARS-CoV-2 infection via TOLLIP-mediated autophagy [60]. SARS-CoV-2-induced increases in ROS cause oxidative stress and mitochondrial electron imbalance. As a result, procaspases, cytochrome C, and pro-apoptotic mechanisms are upregulated, leading to cellular damage and apoptosis [61]. This evidence suggests that ACE2 may be involved in additional pathogenic mechanisms underlying COVID-19 that are not directly associated with inflammation.

Interestingly, serum levels of AT1R and ACE2 autoantibodies are associated with COVID-19, and correlate with the severity of the disease [62,63]. AT1R autoantibodies act as AT1R agonists and aggravate the RAS-mediated pro-inflammatory pathway, while ACE2 autoantibodies act as ACE2 antagonists, thereby functioning as pro-inflammatory factors too [64]. Hence, it has been hypothesized that these autoantibodies may aggravate the inflammatory cascade mediated by RAS in COVID-19 patients, resulting in worse clinical outcomes. Importantly, the plasma of patients with a history of COVID-19 and ACE2 autoantibodies displays reduced activity of the endogenous soluble ACE2 and inhibits the activity of exogenous ACE2. Based on this evidence, it has been hypothesized that the development of ACE2 autoantibodies in COVID-19 patients may upregulate RAS and result in a pro-inflammatory state [64]. This process may underlie the association between COVID-19 and other inflammatory chronic conditions, including neurodegenerative diseases.

## 4. The Relationship between ACE2 and PD: Exploring the Underlying Mechanisms

PD is characterized by the progressive loss of dopaminergic neurons in the substantia nigra pars compacta and the deposition of Lewy bodies and Lewy neurites containing alpha-synuclein [8]. According to Braak staging, at prodromal stages of PD, Lewy pathology initially appears in the olfactory bulbs and the dorsal motor nucleus of the vagal nerve, possibly associated with hyposmia and constipation, respectively. Then, Lewy pathology may spread throughout various brain regions in a stereotypical manner, while it affects dopaminergic neuronal cells in the substantia nigra several years later [65]. Although the pathogenesis of PD remains elusive, several pathophysiological mechanisms, including oxidative stress, neuroinflammation, mitochondrial dysfunction, abnormal protein aggregation and spreading, autophagy dysregulation, impaired apoptotic mechanisms, and gut microbiome imbalance, are implicated in its development [66,67,68,69]. Increased levels of tumor necrosis factor alpha (TNF) and interleukin 1β (IL-1-β) have been related to increased PD risk [70]. Caspases upregulation, NF-κB upregulation, ROS production, and mitochondrial electron imbalance are implicated in PD [6,61]. Inflammasomes, and especially the NLR family pyrin domain containing 3 (NLRP3) inflammasome, are majorly implicated in the elimination of damaged cells and pathogens by microglia. Alpha-synuclein can activate NLRP3, and its upregulation is implicated in PD pathogenesis [6].

ACE2 is expressed in several brain regions, mainly in the thalamus, inferior olivary nuclei, and cerebellum [71], but also in the hippocampus, amygdala, visual cortex, and striatum [5]. Excessive RAS activation in the brain has been associated with oxidative stress, inflammation, immune dysregulation, and abnormal cell growth and proliferation [17,72]. AT1R overactivation upregulates NADPH-oxidase complex 2 (Nox2), resulting in the production of reactive oxygen species (ROS) [73]. Angiotensin II/AT1R/Nox4 pathway-induced oxidative stress is associated with dopaminergic degeneration [74]. In mouse models of age-dependent cardiomyopathy, ACE2 deficiency can increase angiotensin II-induced oxidative stress, inflammation, and neutrophilic infiltration via the AT1R [75]. Exosome-mediated transfer of ACE2 can increase endothelial cell survival [76], and ACE2 modulates mitochondrial function in mice [77]. Given the fact that dysregulation of cell survival, mitochondrial function, inflammation, and excessive oxidative stress are implicated in the pathogenesis of PD, it has been proposed that ACE2 may play a role in this case too.

Accumulating evidence suggests the potential implication of autoimmunity in the development and progression of PD, and autoantibodies targeting the extracellular region of glial or neuronal proteins receive increasing interest [78]. In this regard, a recent study demonstrated that ACE2 and AT1R autoantibodies were increased in the serum of PD patients compared to controls. The levels of AT1R autoantibodies were also associated with various cytokines in this study, including tumor necrosis factor ligand superfamily member 14 (TNFSF14) and 27-hydroxycholesterol. In addition, the levels of both autoantibodies were increased in the serum and cerebrospinal fluid of 6-OHDA-induced rat models of PD. The levels of TNFSF14 and the activity of transglutaminase were also elevated in the substantia nigra of the rat models of PD in this study. Delivery of AT1R autoantibodies in cell cultures could promote dopaminergic neuronal cell death and increase pro-inflammatory cytokine levels. This effect was suppressed by the use of candesartan, an AT1R antagonist [64].

**Figure 1:**
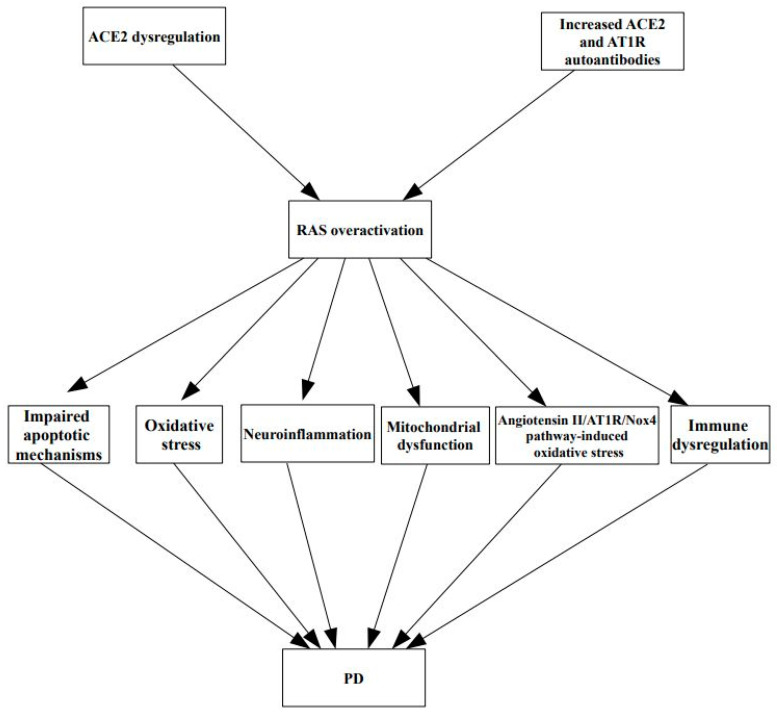
The relationship between the dysregulation of ACE2 and PD. RAS and ACE2 dysregulation play a critical role in PD pathogenesis by modulating apoptosis, mitochondrial function, oxidative stress, neuroinflammation, and autoimmunity. Since these mechanisms are also involved in COVID-19 pathophysiology, it can be hypothesized that ACE2 represents a key molecule connecting these two conditions. ACE2: angiotensin converting enzyme 2; AT1R: angiotensin II type 1 receptor; Nox4: NADPH oxidase 4; PD: Parkinson’s disease; RAS: renin angiotensin system.

Figure 1 illustrates the relationship between the dysregulation of ACE2 and PD.

## 5. ACE2 in PD and COVID-19: Connecting the Dots

Various factors, including environmental toxins, pathogens, tissue injury, and protein aggregation in the brain, may trigger the upregulation of innate immunity, primarily via the activation of microglia. Excessive neuroinflammation, blood–brain barrier dysfunction, alpha-synuclein aggregation, mitochondrial dysfunction, hypoxia, and microvascular damage have been postulated to contribute to post-viral parkinsonism [79]. The sustained activation of this inflammatory response may drive a pro-inflammatory milieu, which could contribute to neurodegeneration [6].

**Figure 2:**
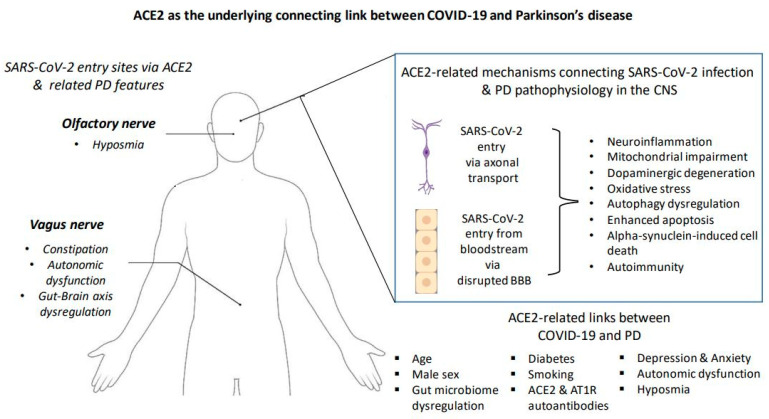
ACE2 as the underlying connecting link between COVID-19 and Parkinson’s disease. Proposed ACE2 mechanisms underlying the relationship between COVID-19 and Parkinson’s disease: SARS-CoV-2 enters the human host cells via its essential cellular receptor, ACE2. ACE2 is located in several tissues, including the lungs, the olfactory epithelium, and the intestinal wall. SARS-CoV-2 can enter the brain through (1) the olfactory and vagus nerve via retrograde axonal transport or (2) the bloodstream via a disrupted blood–brain barrier. ACE2 is also located in the epithelial cells of the blood–brain barrier, where it could facilitate the entry of SARS-CoV-2 into the brain. ACE2 may be implicated in several pathophysiological mechanisms underlying PD pathogenesis, including neuroinflammation, mitochondrial dysfunction, dopaminergic degeneration, oxidative stress, autophagy dysregulation, enhanced apoptosis, alpha-synuclein-induced cell death, and autoimmunity, such as the generation of ACE2 and AT1R autoantibodies. SARS-CoV-2 may create a pro-inflammatory microenvironment in the brain, which may in turn promote neuronal degeneration. Neuroinflammation and degeneration may be initiated in the olfactory bulb and dorsal motor nucleus of the vagus nerve in the brainstem and then reach the dopaminergic neurons in the substania nigra pars compacta. This process follows the Braak stages. Potential ACE2-related links include older age, male sex, gut microbiome dysregulation, diabetes, smoking, depression and anxiety, autonomic dysfunction, and hyposmia [65,80,81,82,83,84,85,86].

ACE2 is highly expressed in many brain regions, including the striatum, and SARS-CoV-2 has been shown to infect neuronal cells. Lesions in the basal ganglia have also been reported in COVID-19 thromboembolic encephalopathy [80]. As a result, it has been proposed that the virus could enter the brain hematogenously or via axonal transport via the olfactory or vagus nerves [81], which are also the initial sites of Parkinson’s disease-related Lewy pathology according to Braak staging [65]. Previous studies in mice showed that the influenza A virus could be transmitted from the respiratory tract to the basal ganglia via the vagus nerve [82]. SARS-CoV-2 is also present in neuronal cells of the myenteric plexus [83]. Hence, it is possible that SARS-CoV-2 may also follow this route through ACE2. Hyposmia and constipation are well known symptoms of prodromal PD; ACE-2 is expressed in nasal goblet and ciliated cells, as well as in the intestinal epithelium [84], further strengthening this hypothesis. SARS-CoV-2 could enter the nasal cavity, then the olfactory bulb and the piriform cortex, and finally the brainstem, where the substantia nigra pars compacta is located [84]. Because the blood–brain barrier is absent in the olfactory bulb, viral entry is made easier [14]. A recent study revealed that COVID-19 infection was associated with reduced gray matter thickness in the parahippocampal gyrus and orbitofrontal cortex, as well as changes in brain areas with a functional connection to the primary olfactory cortex [85]. These alterations suggest tissue injury, and it could be speculated that the spreading of inflammatory and degenerative processes via the olfactory bulb to other PD-related brain regions might be possible [14] (Figure 2).

**Figure 3:**
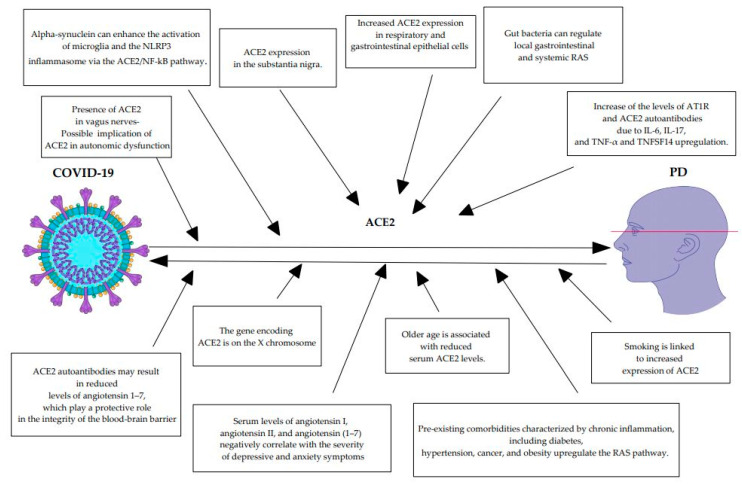
Specific pathways involved in ACE2 dysregulation as a connecting link between COVID-19 and PD. Parts of the figure were drawn by using pictures from Servier Medical Art. Servier Medical Art by Servier is licensed under a Creative Commons Attribution 3.0 Unported License (https://creativecommons.org/licenses/by/3.0/ (accessed on 28 January 2023)). ACE2: angiotensin converting enzyme 2; AT1R: angiotensin II type 1 receptor; COVID-19: coronavirus disease 019; IL-6: interleukin-6; IL-17: interleukin-17; NLRP3: NLR family pyrin domain containing 3; NF-kB: nuclear factor kappa B; PD: Parkinson’s disease; RAS: renin angiotensin system; TNF-α: tumor necrosis factor-α; TNFSF14: tumor necrosis factor superfamily member 14.

The COVID-19 pandemic offers an important opportunity to examine how viral infections might aggravate PD-related neurodegeneration [82]. Table 1 summarizes the mechanisms underlying the aggravation of PD-related neurodegenerations due to SARS-CoV-2 [86,87,88,89,90,91,92,93,94,95,96,97,98,99,100,101,102,103,104,105,106,107,108,109,110,111,112,113,114,115,116,117,118,119,120,121] and Figure 3 summarizes the specific pathways involved in ACE2 dysregulation as a connecting link between COVID-19 and PD.

## 6. Therapeutic Implications

Cardiovascular diseases, such as hypertension and heart failure, have been associated with COVID-19 aggravation. Several ACE inhibitors that are currently used in clinical practice for cardiovascular disorders, including lisinopril and captopril, do not affect ACE2 activity [21]. However, the therapeutic use of ACE inhibitors or angiotensin II receptor blockers in heart failure can restore, and therefore elevate, tissue ACE2 expression [122]. Given this evidence, it has been hypothesized that the use of ACE inhibitors or angiotensin II receptor blockers might increase COVID-19 infection risk. However, large clinical studies have demonstrated that the use of any type of these drugs is not associated with an increased risk of SARS-CoV-2 infection [123] and might actually aid in the suppression of COVID-19 aggravation [124]. Interestingly, the use of ACE inhibitors and angiotensin II receptor blockers has been associated with a potential protective effect on the cognitive impairment of PD patients with hypertension [125]. Moreover, ACE inhibitors and angiotensin II receptor blockers may reduce levodopa-induced dyskinesia occurrence in PD patients with hypertension [126], and the use of ACE inhibitors has been linked to a reduced number of falls in these patients [127]. Of note, the use of RAS inhibitors has been significantly associated with a lower risk of PD according to a large recent study of patients with ischemic heart disease [128].

ACE2 gene therapy or the delivery of recombinant proteins have been proven effective in animal models of atherosclerosis, hypertension, and diabetic nephropathy [128,129,130,131]. In silico analysis has also revealed two substances that activate and increase the activity of ACE2: resorcinolnaphthalein and a xanthenone [132]. Currently, ongoing clinical trials use soluble ACE2 recombinant protein as a decoy for the neutralization of SARS-Cov-2, thereby inhibiting its cellular entry into the host cells and subsequent infection [29,133]. AVE0991, an angiotensin (1–7) analogue, could inhibit age-related neuroinflammation in mouse models of accelerated aging [134], and RAS inhibitors display a neuroprotective potential in dopaminergic neurons via mitochondrial restoration [135]. However, given the complex interplay between ACE2 and neurodegenerative diseases, including PD, the specific effects of ACE2-related treatment approaches against COVID-19 in this subpopulation need to be investigated. 

As mentioned above, NLRP3 inhibition could block the SARS-CoV-2-induced and alpha-synuclein-enhanced activation of the NLRP3 inflammasome in microglia. As there are currently clinical trials using NLRP3 inhibitors for PD, it can be proposed that these molecules may be beneficial for COVID-19-related neurological symptoms, especially COVID-19-related neurodegeneration [6].

## 7. Future Perspectives

The partially controversial results of the abovementioned studies could be partially explained by the different methodological designs, including the diversities in dose, which could result in inconsistent results and conclusions [38]. In addition, the relationship between SARS-CoV-2 and ACE2 may differ between physiological conditions (i.e., at the beginning of SARS-CoV-2 invasion) and inflammatory conditions in established COVID-19 disease. Alterations in ACE2 levels may represent a compensatory mechanism in the latter case. Importantly, the circulating and paracrine RAS may exert diverse biological effects on different tissues. In general, paracrine RAS has been shown to be more important at the tissue level than circulating RAS [136]. Therefore, in order to elucidate the role of RAS and ACE2 in COVID-19 and neurodegeneration, the cell- and tissue-specific roles of both circulating and paracrine ACE2 should be further explored, at least in the lungs and the brain. 

The existing evidence on the effects of vitamin D in PD and COVID-19 is controversial. ACE2 can be downregulated by vitamin D, while there is also evidence supporting the hypothesis that vitamin D can enhance ACE2 production [137]. The clarification of the role of vitamin D in ACE2 regulation may also shed more light on the relationship between COVID-19 and PD.

Interestingly, a recent study indicated that neuronal ACE2 protein levels were downregulated in the hippocampus, entorhinal cortex, amygdala, basal nucleus, middle frontal gyrus, and visual cortex of the brain of patients with Alzheimer’s disease pathology [5]. Given the key role of ACE2 in SARS-CoV-2 cell invasion, the AD-related alterations in ACE2 expression in various brain areas may affect the susceptibility of these regions to SARS-CoV-2 infection. Furthermore, ACE2 expression is increased in endothelial cells of the white matter of COVID-19 patients, and higher ACE2 levels were correlated with increased severity of neurological symptoms [138]. In this regard, it would be interesting to investigate the expression of the ACE2 protein in specific PD-related brain regions and its relationship to SARS-CoV-2 infection.

Since specific ACE2 gene variants affect ligand-receptor interaction, the role of ACE2 variants in COVID-19 susceptibility and severity has also been investigated. In this regard, the G-allele or GG genotype of the ACE2 rs2285666 has been associated with a higher risk of SARS-CoV-2 infection, severity, and mortality [139]. RAS downregulation via ACE inhibitors or AT1R blockers has shown protective effects against cognitive impairment [140]. A recent clinical study found no associations between cognitive decline in PD and specific single nucleotide polymorphisms (SNPs) in genes encoding angiotensinogen, ATR1, ATR2, and ACE [141]. Furthermore, NLRP3 inflammasome gene variants have been associated with critical disease in COVID-19 patients, especially in men and older patients, as well as patients with diabetes type 2, hypertension, and a higher body mass index [142]. A rare NLRP3 genetic polymorphism has been associated with a reduced risk of PD [143]. However, the role of ACE2 and NLRP3 gene polymorphisms in the relationship between COVID-19 and PD is unknown.

ACE inhibitors and AT1R blockers show beneficial effects against levodopa-induced dyskinesias, a common side effect of long-term levodopa treatment in PD patients [126]. However, the link between COVID-19 and levodopa-induced motor complications in PD is unclear, and the implication of ACE2 in this relationship remains unknown. 

Longitudinal studies that will observe COVID-19 patients for the development of PD, PD dementia, or other neurodegenerative conditions are of paramount importance. There are no longitudinal studies that have shown an increased PD risk post-COVID-19 so far, but only a review of approximately 20 cases of parkinsonism that occurred concurrently with or shortly after a SARS-CoV-2 infection since the beginning of the pandemic [14]. 

In addition, it would be of interest to explore potential associations between sustained COVID-19-related hyposmia or anosmia, gastrointestinal or autonomic symptoms, and the development of PD, as well as the role of ACE2 levels or ACE2 autoantibodies in this relationship. Furthermore, the interaction between age, diabetes, smoking, or other environmental exposures with the COVID-19-related PD risk could be further examined. 

## 8. Conclusions

In conclusion, a mutualistic association between COVID-19 and PD can be proposed [5]. The PD-related pathology characterized by a pro-inflammatory milieu, oxidative stress, immune dysregulation, mitochondrial dysfunction, and ACE2 downregulation could enhance the detrimental effects of the SARS-CoV-2 infection. In addition, SARS-CoV-2 infection could possibly increase future PD risk via the enhancement of neuroinflammation, immune dysregulation, oxidative stress, blood–brain barrier disruption, and ACE2 downregulation. This bidirectional relationship paves the way for future research in order to better understand the molecular mechanisms connecting these two conditions and the development of targeted therapeutic approaches.

## Figures and Tables

**Table 1:** Mechanisms underlying the aggravation of PD-related neurodegeneration due to SARS-CoV-2.

Study	Mechanisms
Angelopoulou et al. [68]	The peripheral SARS-CoV-2-induced release of pro-inflammatory cytokines might activate resident glial cells in the brain or stimulate the entry of peripheral immune cells such as T cells into the brain via specific or non-specific antigens.
Tulisiak et al. [65]	Viral infections may cause synucleinopathy in the brain, according to epidemiological and experimental evidence.
Lodygin et al. [86]	β-synuclein-reactive T cells can cause autoimmune neuronal degeneration in the brains of rats.
Matschke et al. [87]	COVID-19 patients display microglial activation and brain penetration of cytotoxic T-lymphocytes, especially in the brainstem.
Philippens et al. [88]	Lewy body pathology has been identified in macaques infected by SARS-CoV-2.
Kaufer et al. [89]	Alpha-synuclein accumulation has been detected in hamsters after SARS-CoV-2 infection.
Cui et al. [5]	Alpha-synuclein can enhance the SARS-CoV-2-mediated activation of microglia and the NLRP3 inflammasome via the ACE2/NF-kB pathway.
Pavel et al. [80]	SARS-CoV-2 infection causes increased levels of bioenergetic cellular stress. The vulnerable dopaminergic neurons may not be able to address the additional cellular COVID-19-induced stress, which could possibly overcome the degeneration threshold.
Wan et al. [90]	Next-generation sequencing analysis has identified ACE2 expression in the substantia nigra.
Wang et al. [91]	The respiratory and gastrointestinal epithelial cells are key hosts of both microbiota and SARS-CoV-2 targets, where ACE2 and TMPRSS2 are highly expressed.
Zuo et al. [92]	A lower number of beneficial microbes and higher levels of opportunistic pathogenic microbes have been found in COVID-19 patients in their fecal microbiomes compared to healthy controls.
Jaworska et al. [93]	Gut bacteria can regulate local gastrointestinal and systemic RAS, while an RAS imbalance in the intestinal wall may affect microbiota composition and activity.
Rodriguez-Perez et al. [63]	COVID-19 may trigger the development of ACE2 and AT1R autoantibodies.
Lamarca et al. [94] Dhillion et al. [95] Herro et al. [96] Jiang et al. [97] Perlin et al. [98]	Increase of the levels of AT1R and ACE2 autoantibodies due to IL-6, IL-17, and TNF-α and TNFSF14 upregulation.
Fleegal et al. [99] Wu et al. [100] Drelich et al. [101]	ACE2 autoantibodies may result in reduced levels of angiotensin 1–7, which play a protective role in the integrity of the blood–brain barrier. ACE2 is also located in the endothelial cells of the blood–brain barrier.
Albornoz et al. [6]	SARS-CoV-2 can impair the blood–cerebrospinal fluid barrier in human brain organoids, and it is also associated with disruption of the blood–brain barrier in hamster models, suggesting that the virus may be able to penetrate the blood–brain barrier.
Angelopoulou et al. [102] Ebrille et al. [103] Vitale-Cross et al. [104]	Given the presence of ACE2 in vagus nerves and the early involvement of the vagus nerve in PD-related Lewy body pathology, it could be speculated that ACE2 may be implicated in the autonomic dysfunction observed in both PD and COVID-19 infection.
Zhao et al. [105] Rocha et al. [106] Okechukwu et al. [107]	Depression and anxiety are common non-motor manifestations of PD. Serum levels of angiotensin I, angiotensin II, and angiotensin (1–7) negatively correlate with the severity of depressive and anxiety symptoms in PD patients, whereas serum ACE and ACE2 levels do not. ACE2 downregulation has also been associated with anxiety and depression in patients with SARS-CoV-2.
AlGhatrif et al. [108] Angeli et al. [109] Chen et al. [110] Lee et al. [111]	Older age is associated with reduced serum ACE2 levels in humans and animal models. A bioinformatics study indicated that ACE2 levels become lower with age in several tissues, including the nervous system and the blood. It is proposed that age-related ACE2 reduction is at least partially associated with the increased morbidity and mortality of COVID-19 elderly patients.
Lee et al. [111]	Given the fact that the gene encoding ACE2 is on the X chromosome, the higher mortality in men among COVID-19 patients could be at least partially explained by the lower expression of the ACE2 gene in males. Male gender is also linked to a higher risk for PD.
Liu et al. [112] Suzuki et al. [113] Lallai et al. [114] Li et al. [115] Angelopoulou et al. [116] Mappin-Kasirer et al. [117] Chen et al. [118] Lu et al. [119] Tong et al. [120]	Smoking might possibly protect against COVID-19 contraction due to increased expression of ACE2, but it may be associated with worse morbidity and mortality in COVID-19 individuals. Smoking has been associated with a reduced risk of PD in several studies. It would be hypothesized that ACE2 may be implicated in the protective effects of smoking in PD.
Verdecchia et al. [121]	Pre-existing comorbidities characterized by chronic inflammation, including diabetes, hypertension, cancer, and obesity, upregulate the RAS pathway.

ACE2: angiotensin converting enzyme 2; AT1R: angiotensin II type 1 receptor; COVID-19: coronavirus disease 2019; IL-6: interleukin-6; IL-17: interleukin-17; NLRP3: NLR family pyrin domain containing 3; NF-kB: nuclear factor kappa B; PD: Parkinson’s disease; RAS: renin angiotensin system; SARS-CoV-2: severe acute respiratory syndrome coronavirus 2; TMPRSS2: transmembrane serine protease 2; TNF-α: tumor necrosis factor-α; TNFSF14: tumor necrosis factor superfamily member 14.

## Data Availability

Not applicable.
